# Measurement properties of comorbidity indices in maternal health research: a systematic review

**DOI:** 10.1186/s12884-017-1558-3

**Published:** 2017-11-13

**Authors:** Kazuyoshi Aoyama, Rohan D’Souza, Eiichi Inada, Stephen E. Lapinsky, Robert A. Fowler

**Affiliations:** 1 Department of Anesthesia and Pain Medicine, The Hospital for Sick Children, University of Toronto, Toronto, Canada; 2 Intensive Care Unit, Mount Sinai Hospital and University Health Network, University of Toronto, Toronto, Canada; 3 Department of Critical Care Medicine, Sunnybrook Health Science Centre, University of Toronto, Toronto, Canada; 4 Interdepartmental Division of Critical Care Medicine, University of Toronto, Toronto, Canada; 5 Department of Anesthesiology and Pain Medicine, School of Medicine, Juntendo University, Tokyo, Japan; 6 Department of Obstetrics and Gynaecology, Division of Maternal-Fetal Medicine, Mount Sinai Hospital, University of Toronto, Toronto, Canada; 7 Institute of Health Policy, Management and Evaluation, University of Toronto, Toronto, Canada; 8 555 University Ave, Room 2211, Toronto, ON M5G 1X8 Canada

**Keywords:** Systematic review, Comorbidity index, Maternal Health Research, Measurement properties, Charlson comorbidity index, Maternal comorbidity index, Elixhauser comorbidity index

## Abstract

**Background:**

Maternal critical illness occurs in 1.2 to 4.7 of every 1000 live births in the United States and approximately 1 in 100 women who become critically ill will die. Patient characteristics and comorbid conditions are commonly summarized as an index or score for the purpose of predicting the likelihood of dying; however, most such indices have arisen from non-pregnant patient populations. We sought to systematically review comorbidity indices used in health administrative datasets of pregnant women, in order to critically appraise their measurement properties and recommend optimal tools for clinicians and maternal health researchers.

**Methods:**

We conducted a systematic search of MEDLINE and EMBASE to identify studies published from 1946 and 1947, respectively, to May 2017 that describe predictive validity of comorbidity indices using health administrative datasets in the field of maternal health research. We applied a methodological PubMed search filter to identify all studies of measurement properties for each index.

**Results:**

Our initial search retrieved 8944 citations. The full text of 61 articles were identified and assessed for final eligibility. Finally, two eligible articles, describing three comorbidity indices appropriate for health administrative data remained: The Maternal comorbidity index, the Charlson comorbidity index and the Elixhauser Comorbidity Index. These studies of identified indices had a low risk of bias. The lack of an established consensus-building methodology in generating each index resulted in marginal sensibility for all indices. Only the Maternal Comorbidity Index was derived and validated specifically from a cohort of pregnant and postpartum women, using an administrative dataset, and had an associated c-statistic of 0.675 (95% Confidence Interval 0.647–0.666) in predicting mortality.

**Conclusions:**

Only the Maternal Comorbidity Index directly evaluated measurement properties relevant to pregnant women in health administrative datasets; however, it has only modest predictive ability for mortality among development and validation studies. Further research to investigate the feasibility of applying this index in clinical research, and its reliability across a variety of health administrative datasets would be incrementally helpful. Evolution of this and other tools for risk prediction and risk adjustment in pregnant and post-partum patients is an important area for ongoing study.

**Electronic supplementary material:**

The online version of this article (10.1186/s12884-017-1558-3) contains supplementary material, which is available to authorized users.

## Background

Maternal critical illness is a medical condition of pregnant and postpartum women which may result in end-organ injury, morbidity or death; and, generally requires treatment with organ supporting care – mechanical ventilation for respiratory failure, intravenous vasoactive medications for septic shock, or transfusions for peri-partum haemorrhage [1]. Maternal critical illness occurs in 1.2 to 4.7 of every 1000 live births in the United States and is associated with a fetal loss rate of 30% [2–4].

Within developed countries, for every 100 episodes of maternal critical illness [5], one woman will die. While approximately 30–40% of maternal critical illness is preventable [6], the events leading to deterioration and risk of death can be rapid and insidious. Effective monitoring and early intervention for those at risk are not always planned or available. Clinicians, patients and families would therefore benefit from a tool that could predict the risk of maternal mortality from commonly available data, across a range of gestational age in order to assist with decision-making on an appropriate level of monitoring and care. Maternal health research would also be assisted by a tool to help risk-adjust in observational studies among patients with different probabilities of death.

Kirshner and Guyatt [7] suggest a methodological framework for the development of predictive measurement tools for clinical and health services research that considers all potential predictive factors of outcomes and include patient-related and health-system domains. In maternal critical care, maternal age, comorbidities, features of the present illness, physiological status and biological profiles have individually been shown to influence the risk of maternal mortality and morbidity [8, 9]. An ideal predictive tool for maternal mortality would therefore incorporate all these domains, and guide management by indicating which patients require an increased level of observation or care in order to prevent critical illness or death.

Patient characteristics and comorbid conditions are commonly used to describe patient populations and are often summarized as an index or score for the purpose of predicting the likelihood of dying. Such an index can also assist in adjusting potential confounding factors in clinical and health services research when attempting to quantify the association of a particular factor with an outcome such as death. Prior studies have shown inconsistent performance (with respect to discrimination and calibration) of common physiology-based indices for pregnant and post-partum women (e.g. Acute Physiology And Chronic Health Evaluation (APACHE) score, the Simplified Acute Physiology Score (SAPS), the Mortality Prediction Model (MPM) and their adaptations) [10–12]. An increasing volume of maternal health research uses administrative datasets; however, there is no existing systematic review and appraisal of comorbidity indices appropriate for health administrative datasets in maternal health research.

### Objective

The objective was to conduct a systematic review to identify and critically appraise measurement properties of comorbidity indices predicting mortality among pregnant and post-partum women.

## Methods

### Criteria for considering studies for review:

#### Type of studies

Studies eligible for inclusion included prospective and retrospective cohort studies, excluding case-reports, case-series and case-control studies.

#### Participants

Participants were pregnant and postpartum women in general wards and ICUs at acute care hospitals. The postpartum period was defined as within 6 weeks from the date of delivery or fetal demise. Patients in outpatient clinics or emergency rooms, not admitted to hospital, were excluded.

#### Index models (prediction models for clinical outcomes being evaluated in this review) [13]

We focused on comorbidity indices. We exclude models based upon physiology-based indices with vital signs and laboratory results because health administrative data sets rarely have this level of granular detail.

#### Target conditions for predicting mortality

The target conditions were comorbidities of hospitalized pregnant and postpartum women.

#### Reference standards to confirm mortality

Clinical follow-up in hospital, or up to 30 days following discharge to determine survival or death during the index hospital stay in each study. Studies that reported on mortality as a composite outcome with other outcomes (e.g. organ dysfunction) were eligible.

### Data sources and search strategy

We performed a literature search using MEDLINE and EMBASE (1946 to May 2017 in MEDLINE, 1947 to May 2017 in EMBASE) for all publications on comorbidity indices, not including vital signs and laboratory results, in the context of maternal health research, using the search terms associated with “pregnant women” as study participants, terms identifying comorbidity indices (“health status indicators”) and index models of interest and “mortality” as the outcome of index models. The detailed search strategy is provided in Additional file 1: Appendix A. The reference lists of identified relevant articles and also the references that cited identified articles were reviewed through Web of Science. In addition, narrative review articles and book chapters were examined. No language restriction was applied for the literature search.

### Data collection

#### Selection of studies

The two independent reviewers (KA, RD) scanned the titles and abstracts of every record retrieved to determine the studies to be assessed further. If it was clear from the title and abstract that the article was not relevant, according to the above eligibility criteria, the reviewers rejected the record. The full texts of the remaining articles were retrieved. Two reviewers (KA, RD) independently assessed and determined the eligibility of studies. Disagreements were resolved by discussion and, when necessary, a third reviewer (RF) was consulted to assist in adjudicating a final decision.

#### Data extraction

Each study was described by general information (title, journal, year of publication status and study design [prospective or retrospective]), study descriptors (number of included patients, age, subgroups, type of comorbidity index, and stated purpose of the model), follow-up information (outcome including mortality rate) and predictive validity (calibration and discrimination).

### Assessment of methodological quality

The quality of studies was assessed using Hayden’s six-domain risk of bias tool for prognostic studies [14] that has been used widely for methodological quality in systematic reviews of risk prediction models [15–17]. The Hayden guidelines address 6 components: 1) Study participation (definition and description of the study subjects); 2) Study attrition (study loss to follow-up); 3) Prognostic factor measurement (quantitatively measured predictors of interest); 4) Outcome measurement (quantitatively measured outcome of interest); 5) Confounding measurement and adjustment; and, 6) Analysis (calibration and discrimination). Each component comprises items that are scored and summed to measure bias within a particular study [14]. Two reviewers (KA, RF) independently assessed the risk of bias for identified studies.

### Search for measurement properties of identified comorbidity indices

A highly sensitive methodological PubMed search filter was applied for each identified comorbidity index [18]. The title and abstract of all search results were then screened, to ensure each study included pregnant or post-partum women. A full literature review was conducted for all remaining articles after the screening of titles and abstracts.

We organized findings according to De Vet measurement tool [19] *development*, *sensibility*, *reliability* and criterion *validity*. Development: The ‘clinimetric’ properties of the indices were evaluated on the domains of item generation, item reduction and item weighting, as suggested by Feinstein [20]. Sensibility in general refers to the “usefulness” of an instrument; for example, describing the tool’s comprehensiveness, clarity and face validity. A common sensibility assessment framework consists of *purpose, population and setting, face* and *content validity,* and *feasibility* [21]. Face validity is the extent to which a test is subjectively viewed as covering the concept it purports to measure. Content validity refers to the extent to which a measure represents all facets of a given construct (e.g. whether items sufficiently address construct domains of the comorbid conditions). That is to say, content validity refers to what the test actually measures, while face validity refers to what it appears to measure. Feasibility addresses features such as simplicity, training requirements and time for administration or completion. Reliability is defined as consistency or stability of measurement [22]. Criterion validity can be assessed by comparing the performance of an index with another measurement in the same clinical situation [19], ideally with the currently accepted gold standard.

## Results

**Fig. 1:**
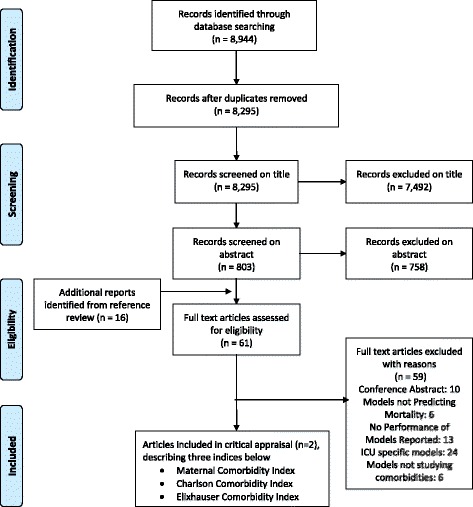
PRISMA flow diagram for MEDLINE, EMBASE

Our initial search retrieved 8944 citations, 8295 of which remained after removal of duplications. Figure 1 represents the study selection process. Screening of the titles and abstracts resulted in 61 relevant articles for which the manuscript full text was assessed for eligibility. From these, 59 studies were excluded, for the following reasons: 10 because there was insufficient information provided in the conference abstract and there was no subsequent full-text article, 6 that did not focus on mortality prediction, 13 that did not report upon model performance, 24 that explored physiology based indices initially developed for non-pregnant populations, in ICU settings, and which employed vital signs and laboratory results in addition to comorbidities (i.e. APACHE, SAPS, the MPM, Sepsis-related Organ Failure Assessment [23–26]), and 6 studies that did not focus on comorbidities. Finally, two eligible articles were identified. One described three comorbidity indices appropriate for health administrative data: the Maternal comorbidity index, the Charlson comorbidity index and the Elixhauser Comorbidity Index [27]. The other article was an external validation of the Maternal Comorbidity Index using Canadian health administrative datasets [28].

### Description of included study and indices

Characteristics of included articles and the three indices are described in Table 1. Bateman and colleagues have recently developed a new measurement tool – the maternal comorbidity index, which predicts an occurrence of maternal end-organ injury or death during the delivery hospitalization through 30 days postpartum, and then validated the index in comparison to the Charlson comorbidity index and the Elixhauser Comorbidity Index [27]. The cohort was derived from the Medicaid Analytic eXtract (a United States-based health administrative dataset). The data set contained 854,823 completed pregnancies and then was separated into a two-thirds development cohort and a one-third validation cohort. The derived index included 20 maternal conditions and maternal age (Additional file 1: Appendix B) [27]. Each condition has a weighted score and the final score is a summation of all conditions.

**Table 1:** Measurement properties of included indices

Study	Year	Design	n	Comorbidity Index	Outcome	C-statistic (95% CI)
Bateman et al. [27]	2013	Retrospective Cohort	854,823	The maternal comorbidity index	Organ injury or death	0.657 (0.647–0.666)
				The Charlson comorbidity index	Organ injury or death	0.578 (0.570–0.585)
				The Elixhauser comorbidity Index	Organ injury or death	0.586 (0.575–0.597)
Metcalfe et al. [28]	2015	Retrospective Cohort	5995	The maternal comorbidity index	Organ injury	0.67 (0.58–0.76)

*CI* Confidence Interval

In the validation cohort, the Maternal Comorbidity Index was assessed for calibration and discrimination properties and was also compared with the Charlson comorbidity index and the Elixhauser Comorbidity Index, using c-statistics and reclassification measure. C-statistics were 0.657 (95% CI 0.647–0.666) in the Maternal Comorbidity Index, 0.578 (95% CI 0.570–0.585) in the Charlson comorbidity index and 0.586 (95% CI 0.575–0.597) in the Elixhauser Comorbidity Index, respectively. The net reclassification improvement (a mechanism to evaluate correct or incorrect classification of risk groups in comparison to the actual outcome group or other indices) for the Maternal Comorbidity Index was 0.118 (*p* < 0.001) when comparing with the Charlson comorbidity index. This means, in comparison with the Charlson comorbidity index, the maternal comorbidity index correctly reclassified 20.8% of women while incorrectly reclassifying only 9.0%, demonstrating improvement in classification for 11.8% of the validation cohort [27, 29, 30].

Thereafter, Metcalfe validated discrimination and calibration properties of the Maternal Comorbidity Index within Canadian health administrative datasets [28]. The main outcome of this validation study was end-organ injury rather than original composite outcome of end-organ injury or death by Bateman. C-statistics for predicting the outcome in hospitalization data was 0.67 (95% Confidence Interval 0.58–0.76). The calibration was high with the Brier score of 0.01 which quantifies the difference between the predicted probability and the actual probability of the outcome, ranging from 0 to 1 (a number closer to 0 has better calibration).

### Methodological quality

The risk of bias of these two articles was assessed according to the Hayden’s guidelines [14], and summarized in Table 2. While the retrospective nature of the identified studies precludes assessment of study attrition, and there remains the potential for unmeasured and unaccounted for confounding variables, overall, the studies have a low risk of bias.

**Table 2:** Risk of Bias in Methodological qualities of identified articles

Study	Study Participation	Study Attrition	Prognostic Factor Measurement	Outcome Measurement	Confounding Measurement and Account	Analysis
Bateman et al. [27]	Low	NA	Low	Low	Moderate	Low
Metcalfe et al. [28]	Low	NA	Low	Moderate	Moderate	Low

*NA* Not Applicable

### Studies for measurement properties

All three comorbidity indices were searched in PubMed, using a methodological PubMed search filter [18], leading to 42, 599 and 34 results for the Maternal, the Charlson and the Elixhauser Comorbidity Indices respectively. Development and sensibility of all three comorbidity indices were assessed on the basis of their original articles [27, 31, 32]. With regard to reliability and validity, we conducted a manual screening of titles and abstracts on the search results in each index. There were no existing studies regarding reliability of all three comorbidity indices in maternal health research. Predictive validity (i.e. discrimination and calibration) of the maternal comorbidity index was investigated in the original article and externally [27, 28].

## Discussion

Our systematic literature search identified only three comorbidity indices that have been directly compared in maternal health research: The Maternal Comorbidity Index, the Charlson Comorbidity Index and the Elixhauser Comorbidity Index. Additional literature search with a methodological PubMed search filter revealed two studies that explored the *validity* of these indices in maternal health research [27, 28]. There is no study assessing *reliability* of these indices in maternal health research. The measurement properties of all three indices are summarized in Table 3 and detailed below.

**Table 3:** Key features of Maternal, Charlson and Elixhauser comorbidity indices

	Maternal Comorbidity Index	Charlson Comorbidity Index	Elixhauser Comorbidity Index
*Development*			
Item generation	Based on clinical relevance and literature, but no consensus method applied	No input from previous index, and no consensus method applied	Based on review of published studies, but no consensus method applied
Item reduction	Odds ratio and p-value from statistical model applied	Adjusted relative risk from statistical model applied, but no clinical relevance for pregnant population	A series of univariable and multivariable regression models
Item weighting	Weights of coefficients from statistical model used	Weights of coefficients from statistical model used	No weighting
*Sensibility*			
Purpose	Predict end-organ injury or death during the delivery hospitalization to 30 days	Predict 1-year mortality	In-hospital mortality
Population	Women who delivered in hospital	General medical patients	Adult, non-maternal inpatients
Setting	Hospitals in U.S.	Medical ward in a hospital	Hospitals in California
Face validity	Exists	Exists	Exists
Content validity	Reasonable	Challenging	Reasonable
Feasibility	No study	Marked training required	No study
*Reliability*	Not studied	Excellent in general No maternal studies	Not studied
*Criterion validity*			
Concurrent validity	Not studied	No maternal studies	No maternal studies

### The maternal Comorbidity index

The Maternal Comorbidity Index was developed from an existing United States database, the Medicaid Analytic eXtract, to predict end-organ injury and 30 day mortality of hospitalized pregnant woman [27]. The included comorbidities are available in Additional file 1: Appendix B. Although potential items in the index were based on clinical relevance and existing literature, no consensus method (e.g. Delphi technique) was used in generating potential items for evaluation and inclusion. Item reduction and weighting were derived on the basis of an odds ratio and coefficients of variables from previously published models predicting outcomes. As a part of a sensibility evaluation, the face validity and content validity was reasonable. Yet, no study has investigated feasibility of applying and incorporating the index into clinical research. C-statistics were near 0.7, indicating acceptable applicability. Although the external validity of the Maternal Comorbidity Index has been investigated [28], subsequent studies investigating reliability have not yet been reported.

### The Charlson Comorbidity index

The Charlson Comorbidity Index was developed on the basis of one-year mortality from a cohort study of 604 patients admitted to an internal medicine service at a New York Hospital during 1 month in 1984 [31]. In the item generation phase, the initial list of 29 potential items was developed based on all comorbidities present in that the cohort. In the item reduction phase, all potential 29 comorbid diseases were investigated in a multi-variable backward proportional hazard model to calculate the adjusted relative risk of one-year mortality using all potential prognostic determinants. The authors excluded determinants with a *p*-value above 0.1 or with an adjusted relative risk below 1.2. Finally, 19 predictors remained in the Charlson Comorbidity Index. The Charlson Comorbidity Index was formulated on the basis of item weighting according to weights of coefficients derived from the mathematical models predicting outcome in nearly exclusively, non-pregnant patients. The components of the weighted index are shown in Additional file 1: Appendix C [31]. The main challenge in the item reduction of the Charlson Comorbidity Index is that it lacks clinical relevance for pregnant patients – the co-morbid conditions common to patients admitted to an internal medicine ward have minimal to modest overlap with pregnant or post-partum patients. Also, the development of the Charlson Comorbidity Index did not include quantitative input from experts and clinicians, which has a significant impact on content validity as a part of sensibility evaluation. In addition, there have been no prior studies to investigate the reliability of the Charlson Comorbidity Index among pregnant patients. Finally, the index showed poor discriminative ability with c-statistics less than 0.6 among pregnant women [27].

### The Elixhauser Comorbidity index

The Elixhauser Comorbidity Index was developed using a California-based population dataset of adult, non-maternal inpatients from 438 acute care hospitals in 1992 [32]. The dataset included 1,779,167 patients and 41 comorbidities were listed. No consensus method was employed for making a list of potential candidates for the comorbidity index. Outcomes were defined as length of stay, charges and in-hospital mortality. Statistically unrelated comorbidities to the outcomes were excluded after a series of univariable and multivariable analyses, leading to a final set of 30 comorbidities (Additional file 1: Appendix D) [32]. The authors did not employ item weighting in the phases of development. The Elixhauser Comorbidity Index was developed mainly for use with administrative data. The index has good face validity as well as content validity as the items are mutually exclusive. Feasibility of using the index was not examined in maternal or related research. In the context of maternal health research, the reliability and criterion validity of the Elixhauser Comorbidity Index have not been elucidated. Lastly, discriminative ability of the index among pregnant women was poor (c-statistic <0.6) [27].

There have been an increasing number of ‘big data’ studies using health administrative datasets in medicine. However, there are many pitfalls in undertaking such studies, most commonly related to unmeasured or unaccounted for confounding in the relationship between exposures and outcomes. Because the severity of patients’ medical conditions is a very common source of potential confounding, risk adjustment among patients with different severity of illness is essential within health administrative datasets in order to more validly estimate the true association of exposures and outcomes. Despite its modest discriminative performance, the Maternal Comorbidity Index is currently the most reasonable comorbidity index for maternal health research using health administrative datasets.

Overall, no comorbidity indices used in maternal health research employed a consensus method to identify or refine variable choices and this limits the sensibility of all indices. Reliability of these indices has not been investigated in maternal health research. Only two articles have explored predictive validity of the indices among pregnant women, showing that the Maternal Comorbidity Index appears better able to predict maternal mortality in comparison with the Charlson and Elixahuser Comorbidity Index [27, 28].

Our review has limitations. First, we did not determine how often these comorbidity indices are used in daily clinical practice, or in maternal health research. Instead, we have identified all available indices in the field and their related measurement properties with a methodological PubMed search filter. Second, there is no standard method to report risk of bias in studies of prediction models. We employed the Hayden’s framework for our study, but the Prediction model study Risk Of Bias Assessment Tool (PROBAST) [33] could be an alternative to assess risk of bias in these studies that will soon be available and may provide an improved approach to bias description.

## Conclusion

Based on this systematic review of the existing literature, the Maternal Comorbidity Index appears to be the most appropriate comorbidity index for use with health administrative datasets of pregnant or post-partum women; however, it requires further appraisal of feasibility and reliability, in order to better appreciate its ability to perform outcome prediction and risk adjustment. Also, given the modest discrimination of the Maternal Comorbidity Index, evolution of this and other tools for risk-adjustment in pregnant and post-partum patients will be an important area for ongoing study.

## Additional file

Additional file 1: Appendix A Search strategy in MEDLINE, EMBASE. Detailed search strategy we used in MEDLINE and EMBASE. **Appendix B** Weighted conditions of the Maternal Comorbidity Index. Detailed description of the Maternal Comorbidity Index. **Appendix C** Weighted conditions of the Charlson Comorbidity Index. Detailed description of the Charlson Comorbidity Index. **Appendix D** List of comorbidities in the Elixhauser Comorbidity Index. Detailed description of comorbidities in the Elixhauser Comorbidity Index. (PDF 58 kb)

## Abbreviations

CI: Confidence Interval
ICU: Intensive Care Unit
