# Supplementary material for: Measurement properties of comorbidity indices in maternal health research: a systematic review
Source: BMC Pregnancy Childbirth. 2017 Nov 13;17:372. doi: 10.1186/s12884-017-1558-3 (PMC5683518; doi:10.1186/s12884-017-1558-3)
Supplement: Additional file 1: Appendix A — Search strategy in MEDLINE, EMBASE. Detailed search strategy we used in MEDLINE and EMBASE. Appendix B Weighted conditions of the Maternal Comorbidity Index. Detailed description of the Maternal Comorbidity Index. Appendix C Weighted conditions of the Charlson Comorbidity Index. Detailed description of the Charlson Comorbidity Index. Appendix D List of comorbidities in the Elixhauser Comorbidity Index. Detailed description of comorbidities in the Elixhauser Comorbidity Index. (PDF 58 kb) [file 12884_2017_1558_MOESM1_ESM.pdf]

Appendix A. Search strategy in MEDLINE, EMBASE

Pregnant women

Obstetrics/
Pregnant woman/
Postpartum period/
Perinatal care/
Pregnancy/
Pregnancy, high- risk/
Exp Pregnancy complications/
Delivery, Obstetric/ mo [mortality]
Labor, obstetric/
Obstetric labor complication/

Mortality

Maternal death/
Maternal mortality/

Comorbidity Index

Exp health status indicators to include:
Health status indicators/or apache/or organ dysfunction scores/ or patient acuity/ or "severity of illness index"/ or karnofsky performance statues/ or sickness impact profile/"outcome assessment (health care)"
Forecasting/
Evaluation studies as topic/or program evaluation/ or "reproducibility of results/ or validation studies as topic/
Exp Risk to include: risk/ or logistic models/ or risk assessment/ or risk factors/
Calibration/
decision support techniques/
roc curve/

Keywords

Charlson.tw.
Elixhauser.tw.

Appendix B. Weighted conditions of the Maternal Comorbidity Index

Assigned weights for diseases	Conditions
5	Severe Preeclampsia or Eclampsia
	Chronic Congestive Heart Failure
4	Congenital Heart Disease
	Pulmonary Hypertension
3	Chronic Ischemic Heart Disease
	Sickle Cell Disease
2	Multiple Gestation
	Cardiac Valvular Disease
	Systemic Lupus Erythematosus
	HIV
	Mild or Unspecified Preeclampsia
	Drug Abuse
	Placenta Previa
1	Chronic Renal Disease
	Pre-existing Hypertension
	Previous Cesarean Delivery
	Gestational Hypertension
	Alcohol Abuse
	Asthma
	Pre-existing Diabetes Mellitus
3	Maternal Age (y), Older than 44
2	40-44
1	35-39

Appendix C. Weighted conditions of the Charlson Comorbidity Index

Assigned weights for diseases	Conditions
6	Metastatic Solid Tumor
	AIDS
3	Moderate or Severe Liver Disease
2	Hemiplegia
	Moderate or Severe Renal Disease
	Diabetes with End Organ Damage
	Any Tumor
	Leukemia
	Lymphoma
1	Myocardial Infract
	Congestive Heart Failure
	Peripheral Vascular Disease
	Dementia
	Chronic Pulmonary Disease
	Connective Tissue Disease
	Ulcer Disease
	Mild Liver Disease
	Diabetes

Appendix D. List of comorbidities in the Elixhauser Comorbidity Index

1. Congestive Heart Failure	16. AIDS
2. Cardiac Arrhythmias	17. Lymphoma
3. Valvular Disease	18. Metastatic Cancer
4. Pulmonary Circulation Disorders	19. Solid Tumor without Metastasis
5. Peripheral Vascular Disorders	20. Rheumatoid Arthritis/ Collagen Vascular Diseases
6. Hypertension	21. Coagulopathy
7. Paralysis	22. Obesity
8. Other Neurological Disorders	23. Weight Loss
9. Chronic Pulmonary Disease	24. Fluid and Electrolyte Disorders
10. Diabetes, uncomplicated	25. Blood Loss Anemia
11. Diabetes, complicated	26. Deficiency Anemia
12. Hypothyroidism	27. Alcohol Abuse
13. Renal Failure	28. Drug Abuse
14. Liver Disease	29. Psychoses
15. Peptic Ulcer Disease excluding Bleeding	30. Depression
